# Effects of Self-Enhancement on Eye Movements During Reading

**DOI:** 10.3389/fpsyg.2019.00343

**Published:** 2019-02-25

**Authors:** Ya Lou, Huajian Cai, Xuewei Liu, Xingshan Li

**Affiliations:** ^1^CAS Key Laboratory of Behavioral Science, Institute of Psychology, Beijing, China; ^2^Department of Psychology, University of Chinese Academy of Sciences, Beijing, China; ^3^Beijing Institute of Education, Beijing, China

**Keywords:** self enhancement, reading, eye movements, language sciences, personality and social psychology

## Abstract

Previous studies show that readers’ eye movements are influenced by text properties and readers’ personal cognitive characteristics. In the current study, we further show that readers’ eye movements are influenced by a social motivation of self-enhancement. We asked participants to silently read sentences that describe self or others with positive or negative traits while their eyes were monitored. First-fixation duration and gaze duration were longer when positive words were used to describe self than to describe others, but there was no such effect for negative words. These results suggest that eye movements can be influenced by the motivation of self-enhancement in addition to various stimuli features and cognitive factors. This finding indicates that the eye movement methodology can potentially be used to study implicit social cognition.

## Introduction

We move our eyes three to four times per second when we are awake to selectively perceive visual information that is most salient or most relevant to our current task (Rayner, 1998, 2009). Decades of eye movement research has shown that our eye movements are influenced by various features of visual stimuli (e.g., words frequency in reading) (Rayner, 1998, 2009) and diverse personal cognitive characteristics (e.g., reading skills) (Rayner and Reichle, 2010; Kuperman and Dyke, 2011). For example, high-frequency words are typically fixated for less time and skipped more often than low-frequency words (Inhoff and Rayner, 1986; Rayner and Duffy, 1986); skilled readers make shorter fixations on words, longer forward saccades (Jared et al., 1999; Ashby et al., 2005), and fewer regressions compared to less-skilled readers (Ashby et al., 2005). Moreover, the eye movements also vary with purposes. For example, Kaakinen et al. (2002) examined the effects of reading goals on eye movement behavior. The reading goal was induced by instructing the participants to imagine that they were going to live in another country. Then the participants were asked to read an expository text that included four remote countries. They found that readers made more and longer fixations on sentences that described the conditions of that country than on other sentences. The current research aims to explore whether eye movement in reading could be influenced by a special human motivation, that is, the desire for a positive self or self-enhancement.

Word processing time might also be affected by high-level cognition such as motivation. For example, self-enhancement, a type of motivation that works to make people feel good about themselves, might affect eye movements during reading. Self-enhancement makes people favor positive over negative self-views (Sedikides and Gregg, 2008). A positive self is significant for physical and mental health (Taylor and Brown, 1988), well-being (Baumeister et al., 2003), and coping with threats and life difficulties (see Alicke and Sedikides, 2009 for a review). Self-positivity may manifest on various behavioral indexes, such as trait endorsement (D’Argembeau et al., 2005; Kwan et al., 2007), reaction time (Paulhus and Levitt, 1987; Gebauer et al., 2012), and neural responses, such as electroencephalograph (EEG) signal (Luck, 2005; Cai et al., 2016; Hampton and Varnum, 2017). Therefore, self-enhancement might influence people’s attention allocation during reading, favoring positive information about self.

**FIGURE 1:**
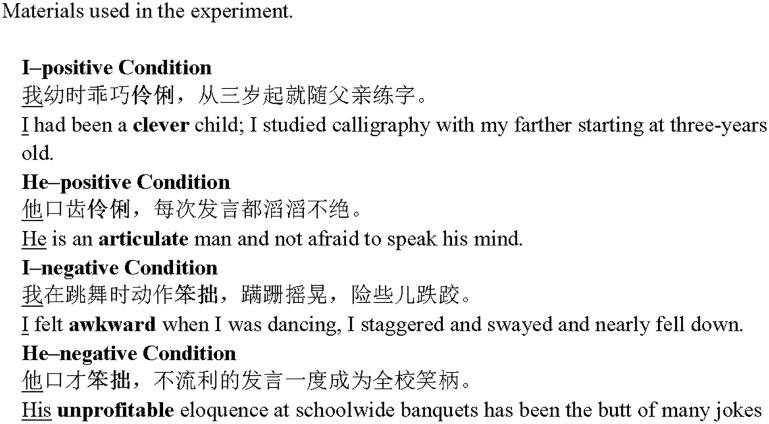
Materials used in experiment. The identify words are underlined and the trait words are in bold letters for the purpose of illustration (the characters were neither underlined nor made bold in the experiment).

This research explored whether self-enhancement manifests in eye movements in reading, or whether self-enhancement influences eye movements in reading self-relevant information. Accordingly, the participants were asked to silently read sentences that describe the self or others with positive or negative traits while their eyes were monitored. Each sentence contained one identity word (i.e., I or He) and one attribute word (i.e., positive or negative) (see Figure 1 for examples). Previous studies showed that people tend to judge positive personality attributes to be more appropriate in describing themselves than in describing others and therefore self-enhancement may encourage people to “elaborate, dwell on” positive self-evaluative information (Heine et al., 1999, p. 760). We inferred that positive traits that describe the self may obtain longer fixation time than those describing the other person (i.e., *he*).

## Materials and Methods

### Participants

A total of 40 undergraduate students from Beijing Forestry University and China Agricultural University participated in this experiment. Three participants were excluded because of technical problems or track loss during eye movement recording. Participants provided consent in accordance with the protocols approved by the ethics committee of Institute of Psychology, Chinese Academy of Sciences.

### Apparatus

Eye movements were recorded using an SR-Research Eyelink 1000 eye tracker (Kanata, ON, Canada) sampling at a rate of 1,000 Hz. Eye movements were recorded from the right eye during binocular viewing. The sentences were displayed as a single line of text using 24 point Song font. The participants were seated at a distance of 58 cm from the computer monitor.

### Materials and Design

The trait words were adapted from a previous study (Cai, 2003), and comprised 12 positive words and 12 negative words (see Table 1). The average frequency of the positive trait words (*M* = 45.53 occurrences per million words, *SD* = 56.69) was higher than that of the negative ones (*M* = 8.59 occurrences per million words, *SD* = 10.95). Each trait word was embedded in two different sentence frames with the following subjects in the sentences: one with the embedded word “I” preceding the trait word and the other with “He.” The word “I” and “He” were used as identity words. Therefore, 12 sentences were created for each of the following four conditions: *I–positive, He–positive, I–negative*, and *He–negative* (see Figure 1). The average sentence length ranged from 16 to 30 characters with a mean of 20.85 characters and a standard deviation of 3.45. The same number of sentences was created as filler sentences in which neither identity nor trait word was included.

**Table 1:** Trait words used in this experiment.

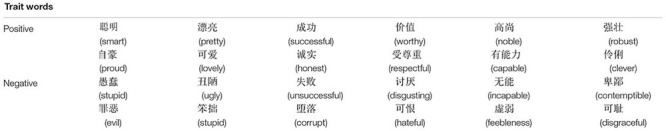

### Procedure

The participants were tested individually. When they arrived at the lab, they were informed that this experiment was designed to use an eye tracking technology to investigate sentence-comprehension processes. However, they were unaware of the experiment’s purpose. Thereafter, they performed a calibration procedure by looking at a sequence of three fixation points that were randomly displayed horizontally across the middle of the computer screen. The maximal calibration error was 0.5°. Calibration was conducted at the beginning of the experiment and was conducted again during the experiment when necessary. At the beginning of each trial, a drift check was conducted to ensure that the error of the eye tracker was within the allowable range. Thereafter, the participants looked at a square located at the position of the first character of the sentence. After they fixated at this square for 0.5 s, the entire sentences appeared. The participants silently read the sentences, and they were required to press a button when they had completed reading these sentences. A comprehension question with a two-alternative forced-choice response was asked after each of all the 24 filler items and participants responded by pressing one of two keys on a response box. These questions were created to ensure that the participants carefully read the sentences. The mean accuracy of the comprehension questions was 95%, thereby indicating that the participants carefully read the sentences.

### Data Analysis

Fixations above 1000 ms or below 80 ms were excluded from analyses. We report the following eye movement measures for the target words in the sentences (Rayner, 1998): (a) First-fixation duration (duration of the first first-pass fixation on the target word), (b) Gaze duration (sum of all first-pass fixations on the target word prior to proceeding to another word), (c) Skipping probability (the probability that the target word was skipped on first-pass reading), and (d) Total reading time (sum of all fixations on the target word, including regression). First-fixation duration and gaze duration are sensitive to early processing associated with lexical identification, whereas total reading times are sensitive to later processes associated with integration (Inhoff, 1984). Table 2 presents the descriptive statistics of these eye movement measures.

**Table 2:** Eye movement measures in the trait word region.

	Positive			Negative		
	I		HE		I		HE	
First-fixation duration	231	(5)	214	(5)	226	(5)	233	(6)
Skipping probability	0.28	(0.02)	0.33	(0.02)	0.23	(0.02)	0.25	(0.02)
Gaze duration	284	(12)	257	(10)	259	(8)	261	(8)
Total reading time	368	(15)	373	(15)	332	(12)	334	(12)
Regression in	0.12	(0.02)	0.12	(0.02)	0.10	(0.01)	0.10	(0.01)


*First-fixation duration, gaze duration, and total time were measured in ms. SEs are provided in parentheses.*

Given that high-frequency words are processed faster than low-frequency words (known as frequency effect) (Rayner and Duffy, 1986), and the frequency of the positive trait words were higher than those of the negative trait words, and the comparison between negative and positive words was not relevant to our research question, we did not directly compare eye movement measures between the negative words and positive words. Instead, the key comparisons were the results between the *I–positive* and *He–positive* conditions and between the *I–negative* and *He–negative* conditions.

Eye movement data were analyzed using linear mixed-effects models (LMM) for continuous variables (Baayen et al., 2008; Jaeger, 2008), in which the participants and items were considered as random effects. Identity words, trait words, and their interactions were entered as fixated effects. The analyses were performed using the *lme4* package (Bates et al., 2014) in the R statistical software (Version 3.3.1, R Core Team, 2016), and the lmerTest Package was used to get the *p*-value for tests for fixed effects.

## Results

### First-Fixation Duration

First-fixation durations were shorter in the positive condition (*M* = 223 ms, *SE* = 4) than in the negative condition (*M* = 229 ms, *SE* = 4), *b* = -22.575, *SE* = 10.279, *t* = -2.196, *p* = 0.03. No difference was observed between the identity conditions (the *I* condition: *M* = 228 ms, *SE* = 4, the *He* condition: *M* = 224 ms, *SE* = 4), *t* = -1.410, *p* = 0.158. However, the interaction effect between the trait valence and identity was significant, *b* = 28.591, *SE* = 10.516, *t* = 2.719, *p* < 0.01.

Planned comparisons showed that first-fixation durations in the *He-positive* condition (*M* = 214 ms, *SE* = 5) were shorter than those in the *I-positive* condition (*M* = 231 ms, *SE* = 5), *b* = 17.795, *SE* = 7.136, *t* = 2.494, *p* = 0.01. First-fixation duration on the negative trait words did not differ between the *He–negative* condition (*M* = 233 ms, *SE* = 6) and *I–negative* condition (*M* = 231 ms, *SE* = 5), *t* = -1.2, *p* = 0.2.

### Gaze Duration

The interaction effect of the trait words and identity was not significant, *b* = 34.407, *SE* = 18.207, *t* = 1.890, *p* = 0.06. No main effect of the trait word and identity was observed, both *t* < 1. Since the interaction was close to significant, we also conducted some further exploratory analyses. Planned comparisons showed that gaze durations were shorter in the *He–positive* (*M* = 258 ms, *SE* = 10) than those in the *I–positive* (*M* = 284 ms, *SE* = 12) condition, *b* = 27.56, *SE* = 14.31, *t* = 1.93, *p* = 0.05. Gaze duration on the trait words did not differ between the *He–negative* (*M* = 261 ms, *SE* = 8) and *I–negative* condition (*M* = 259 ms, *SE* = 8), *t* < 1. The pattern of gaze duration replicated the results from the first-fixation duration.

### Other Measures

Neither the main effects of the trait words, identity, nor the interaction were significant for the other measures (skipping probability, total time, all *t* < 1).

## Discussion

This study analyzed whether human motivation, particularly self-enhancement, influences eye movements during reading. The identity words (i.e., I versus He) and trait words (i.e., positive versus negative) were embedded in the sentences. Accordingly, four sentence conditions (i.e., *I–positive*, *He–positive*, *I–negative*, and *He–negative*) were created. As expected, we found that first-fixation duration and gaze duration in the *I–positive* condition were longer than those in the *He–positive* condition. However, we found no difference in fixation time on negative words.

These findings showed that self-enhancement can affect eye movement behavior during reading. To enhance or maintain a positive self, people often selectively remember their strengths rather than weaknesses. One way to do this is at the encoding stage of memory through selective attention. As a result, people dwell longer on positive words that describe self in reading. These results suggest that eye movements are affected by reading-related factors (e.g., reading material features and reading ability) and human motivation (e.g., self-enhancement), thereby extending our understanding of the range of factors that can affect mechanisms of eye movement control during reading.

For negative traits, we did not observe shorter fixation time on negative words that describing *I* than those that describe *he*. Two factors might have jointly affected the processing of negative words. First, negative words that describing *I* might be processed for shorter time than those that describe *he* due to the need for self-protection. However, there may be considerably longer fixation time because negative self-information may constitute a conflict with the existing self-positivity, thereby attracting further attention due to its inconsistency or novelty. These two opposite factors might have caused a small difference (or no difference) in fixation time between the two *negative* conditions.

In addition to enhancing our understanding of eye movement control during reading, our findings also suggest that eye movement methodology can be used to study the on-line effects of self-positivity during comprehension. Previous studies have used self-report scales (Rosenberg, 1965), reaction time task (e.g., Implicit Association Test or IAT, Greenwald et al., 1998), and electroencephalograph EEG signal (Luck, 2005; Wu et al., 2016) to measure self-positivity. Compared with other methodologies, eye tracking technique has a few advantages. First, this technique reflects moment-to-moment cognitive processes without interfering with the natural behavior of the participants. Second, eye movement data provide the researcher with valuable temporal information about exactly when a manipulation exerts influences. Moreover, the task is based on spontaneous reactions, thereby possibly assisting in sidestepping many artifacts, such as social desirability and response styles.

There are some limitations in the current study. First, we only used limited number of stimuli. Further studies are needed to investigate whether the effects observed in the current study can be extended to other types of positive and negative words. Second, we did not directly compare the effects that were observed with eye tracking technology with the findings that were observed using other technologies. Further studies are needed to address these issues.

In summary, we showed that eye movements can be influenced by the motivation of self-enhancement beyond various stimuli features and cognitive factors. This finding broadens our understanding of the sensitivity of eye movements to high level cognitive processes by showing that differences in the processing of self versus other descriptive words are detectable in early processing using eye movement measures.

## Author Contributions

XiL and YL designed the experiments. YL and XuL carried out experiments and analyzed the experimental results. YL, XiL, XuL, and HC wrote the manuscript.

## Conflict of Interest Statement

The authors declare that the research was conducted in the absence of any commercial or financial relationships that could be construed as a potential conflict of interest.
